# HIV/HBV Co-Infections: Epidemiology, Natural History, and Treatment: A Review Article

**Published:** 2011-12-01

**Authors:** R Ranjbar, A Davari, M Izadi, N Jonaidi, S M Alavian

**Affiliations:** 1 Molecular Biology Research Center, Baqiyatallah University of Medical Sciences, Tehran, Iran; 2 Health Research Center, Baqiyatallah University of Medical Sciences, Tehran, Iran; 3 Baqyiatallah Research Center for Gastroenterology and Liver Disease, Baqiyatallah University of Medical Sciences, Tehran, Iran

**Keywords:** HIV, Hepatitis B, Co-infection, Acute, Chronic, Epidemiology

## Abstract

Hepatitis B virus (HBV) infection, one of the major health priorities, accounts approximately for 350 million chronic cases and a global total of 33 million people were living with human immunodeficiency virus (HIV) in the world.Co-infection with HIV and the HBV presents a significant challenge to health care providers, with different prevalence rates in different parts of the world. It is important to screen all HIV infected individuals for HBV infection and reverse. Infection with HBV becomes more violent in patients co-infected with human immunodeficiency syndrome. HIV/HBV co-infected individuals are at increased risk of chronic hepatitis, cirrhosis, and hepatocellular carcinoma, and of experiencing HAART toxicity. In this review, the latest statistics on epidemiology of HIV, HBV and their co-infection has been presented along with prominent characteristics of HBV. Transmission routes which are the common between HBV and HIV are described and the most important ones are described according to the regional and age features. Also, there is a series of actions being performed once HBV infections occur to prevent HIV or to diagnose if the HBV-infected individuals are also infected with HIV. As in treatment case, some of the frequent treatment methods including applying interferon and using nucleoside and nucleotide analogues have been discussed. Finally, we would explain the new recommendations for treating patients who were co-infected with HBV and HIV, including staging HBV and HIV treatment, based on the stage of each disease. It also outlines the optimal treatment options, whether the patient is treated for HBV first, HIV first, or HIV and HBV together.

## Latest Courses in Epidemiology

Hepatitis B, one of the major health priorities, accounts approximately for 350 million chronic cases out of 2 billion people infected worldwide. [1]An estimated 600,000 people die each year because of the acute or chronic consequences of hepatitis B. Human immunodeficiency virus (HIV) is one of the other viruses in charge of infectious diseases. A global total of 33 million (30.6–36.1 million) people were living with HIV from which 2.7 million (2.2–3.2 million) individuals were newly infected in the late 2008 and

2.0 million (1.8–2.3 million) died of AIDS-related illnesses in 2007 (Table 1).[2] Infection with HBV as well as HIV infection is one of the major concerns globally. Co-infection with the HIV and the hepatitis B virus (HBV) presents a significant challenge to health care providers, with different prevalence rates in different parts of the world and in average affecting approximately 10% of persons with HIV infection.[3] HBV and HIV share the common mode of transmission; thus co-infection of these two viruses occurs frequently.[4][5] Despite the high rate of co-infection, the correlation between HBV and HIV mostly depends on the affected community as well as transmission route. In Iran as an instance, HBV alone has been transmitted vertically and most HBV affected individuals were nevertheless HIV negative.[6][7] Routes of transmission can be characterized by the geographical origin of infected patients as in low HBV prevalence ≤2% HBsAg -in USA, Western Europe, Australia, and New Zealand which is mostly caused by drug injection and unprotected sex between adults. In this case, there is a co-infection rate of 5-10% which is 10 times higher than general population. Average prevalence of 2-7% HBsAg- has been reported in Japan, Middle East, Central America, and Latin America. Finally, high HBV endemicity of 8-15% HBsAg exists in Sub-Saharan Africa, South East Asia, and China. Perinatal transmission, close contact with households, and medical or cultural procedures (scarification and tattoos in Africa) are the most common ways of viral spread in second group in which 10-20% corresponds for the co-infection.[8][9]

**Table 1:** HIV and AIDS prevalence in various regions, a comparison between years 2001 and 2008.[19]

****		**Year**	**Adults and children living with HIV**	**Adults and children newly infected with HIV**	**Adult prevalence (%)**	**Adult and child deaths due to AIDS**
High prevalence (8-15%)	Sub-Saharan Africa	2008	22.4 million (20.8-24.01 million)	1.9 million (1.6-2.2 million)	5.2 (4.9-5.4)	1.4 (1.1-1.7)
		2001	19.7 million (18.3-21.2 million)	2.3 million (2.0-2.5 million)	5.8 (5.5-6.0)	1.4 (1.2-1.7)
	South and South-East Asia	2008	3.8 million (3.4-4.3 million)	280000 (240000-320000)	0.3 (0.2-0.3)	270000 (220000-310000)
		2001	4.0 million (3.5-4.5 million)	310000 (270000-350000)	0.3 (<0.3-0.4)	260000 (210000-320000)
Intermediate prevalence (2-7%)	Middle East and North Africa	2008	310000 (250000-380000)	35000 (24000-46000)	0.2 (<0.02-0.3)	20000 (15000-25000)
		2001	200000 (150000-250000)	30000 (24000-46000)	0.2 (0.1-0.2)	11000 (7800-14000)
	Latin America	2008	2.0 million (1.8-2.2 million)	170000 (150000-200000)	0.6 (0.5-0.6)	77000 (66000-89000)
		2001	1.6 million (1.5-1.8 million)	150000 (140000-170000)	0.5 (<0.5-0.6)	66000 (56000-77000)
Low prevalence (<2%)	Western and Central Europe	2008	850000 (710000-970000)	30000 (23000-35000)	0.3 (0.2-0.3)	13000 (10000-15000)
		2001	660000 (580000-760000)	40000 (31000-47000)	0.2 (<0.2-0.3)	7900 (6500-9700)
	North America	2008	1.4 million (1.2-1.6 million)	55000 (36000-61000)	0.6 (0.5-0.7)	25000 (20000-31000)
		2001	1.2 million (1.1-1.4 million)	52000 (42000-60000)	0.6 (0.5-0.7)	19000 (16000-23000)

Moreover in Iran, the prevalence of HBsAg has differed through the time and after mass vaccination in following years, the number of HBsAg positive individuals started to decrease. Recent studies have shown a remarkable decline where the prevalence rate of HBV was 2.14% or generally 1.5 million people all over the country.[10] Hajiani et al. as well as Nokhodian et al. have provided epidemiological data which shows different prevalence ratios in various areas of Iran fluctuating from 1.7% in Fars Province to 5.0% in Sistan and Baluchestan Province.[7][11] A recent study by Seyed Alinaghi in 2010 illustrates that 3% of general population were infected with HBV before the year 1993; it reduced to 1.5-2% in current years, though, mostly because of the medical cares performed by health providers in Iran.[12] Around 40 million people are worldwide infected with HIV.[13] After introduction of highly active antiretroviral therapy (HAART) has led to a decline in death rate from AIDS-related causes and liver disease still appears to be one of the primary causes of morbidity and mortality, though.[14][15] mostly the parts of the world with a high rate of one infection overlap with high prevalence of the other one, that is, infection with one of the viruses is mostly followed by the other one. In case of HBV/HIV co-infection, biological signs of prior HBV infection have been seen in 90% of HIV-infected individuals (defined by the presence of serum anti-HBc Ab) and 5–15% showed chronic infection (defined by the presence of serum HBsAg) all around the world. [16]HBV incidence fluctuated markedly among different HIV-infected populations, but geographical location established different rates. In areas with low HBV endemicity, such as the United States, Australia, and Europe, HBV and HIV usually happened in adulthood either sexually or through percutaneous transmission. Infection with HBV often follows HIV because HBV is nearly 100-fold more likely to be transmitted than HIV. The prevalence of HBV co-infection in such low endemicity areas is 5-7% of HIV-infected individuals but it is affected by route of infection. The highest frequency of coinfection is because of MSM (men who have sex with men), ranging from 9-17%, and the lowest was from heterosexual transmission.[17] Whereas in countries with intermediate and high HBV endemicity, the main routes of HBV transmission were perinatal or in early childhood; so HBV infection usually preceded HIV infection by decades. In these countries, most studies suggested that HBV co-infection prevalence was 10- 20%, but some showed as low as 6%.[18]

Nowadays, around 1/3 of people in the world had a contact history with HBV infection presented with isolated anti-HBcAb during their lives. Among these, 350 million remained infected chronically and became carriers of the virus. Three quarters of the world’s population live in areas where there are high levels of infection.[19] There is a new entity in coinfection of HBV/HIV that the HBs Ag is negative and occult HBV persists. [20][21]The prevalence of OBI in HIV-positive patients varies considerably from 0% to 89.5% in different geographical regions.[21]

## Virological Features

### Classification and Structure

HBV, a prototype member of the Hepadnaviridae (hepatotropic DNA virus) family, is one of the Hepadnaviruses which strongly appeals to infect liver cells, but hepadnaviral DNA can be traced in pancreas, kidney, and mononuclear cells, as well.[22][23] HBV virions are particles with double shells, 40 to 42 nm thick with an outer lipoprotein envelope. The envelope has three envelope glycoproteins (or surface antigens) related to each other. The viral nucleocapsid which is also called core exists within the envelope. Viral genome, a relaxed-circular, partially duplex DNA containing 3.2 kb, and a polymerase responsible for the synthesis of viral DNA in infected cells are included in the core. HBV virus seems to have multiple viral genotypes, according to the data acquired from DNA sequencing of many isolates of HBV, each of which with a characteristic geographic distribution.[24][25][26] Other than virions, cells infected with HBV produce two different subviral lipoprotein particles of spheres and filamentous forms of about 20 nm. Envelope glycoproteins and host-derived lipids are the only parts HBsAg particles and they are usually significantly more than virions in number (1000:1 to 10,000:1).[25]

### Pathogenesis of Hepatitis B

The HBV replication cycle is not directly cytotoxic to cells, as in, many HBV carriers show no symptoms and have minimal liver injury, however intrahepatic replication of the virus continues extensively. Therefore, immune responses to viral antigens expressed by infected hepatocytes are the main signs of hepatocellular injury. Clinical observations also showed that patients who suffered from immune defects and were infected with HBV often had mild acute liver injury but were major chronic carriers.[27][28] The immune responses to HBV and their role in the hepatitis B pathogenesis are still not completely understood. Several clinical studies showed that in acute, self-limited hepatitis B, strong T-cell responses against many HBV antigens were proven to exist in the peripheral blood, including both major histocompatibility-complex (MHC) class II, CD4+ helper T cells and MHC class I, CD8+ cytotoxic T lymphocytes. The antiviral cytotoxic T-lymphocyte response which is against multiple epitopes within the HBV core, polymerase, and envelope proteins, and also strong helper T-cell - against C and P proteins occur in acute infection. By contrast, in chronic carriers of HBV, such virus-specific T-cell responses are greatly reduced, at least as examined in cells obtained from peripheral blood.[29] Following the introduction of highly active antiretroviral therapy (HAART), liver disease is now the major cause of non-AIDS-related deaths in HIV-1-infected patients.[30][31][32] Progression to cirrhosis seems to be more rapid and more common, and liver-related mortality is higher, in HIV/HBV coinfection than with either infection alone.[33]

## Natural History and Transmission Routes

### Hepatitis B Virus

Percutaneous and mucous membrane exposures to infectious blood and body fluids containing blood are the main routes of HBV transmission.[34] Percutaneous exposure includes blood or blood products transfusion, using contaminated equipment in therapeutic injections and other health-care related practices, injectable drug use, and needle sticks or other injuries from sharp instruments occurred by hospital staff.[35][36] Perinatal and sexual exposures to HBV are two other modes of transmission which are extremely efficient. HBV can also spread among household through contacts with a chronically infected person.[37][38] Moreover, tattooing and acupuncture have been in charge of some occasional outbreaks.[36] Chronic HBV infection is considered as two distinct phases: HBeAg positive and HBeAg negative.[39] In most cases, transmissions result from non-compliance with aseptic techniques and recommended infection control procedures that were designed to prevent post-practice infections due to cross-contamination of medical equipment and devices. In developing countries, donor testing for HBsAg is not common and, unsafe therapeutic injection practices, including inadequately sterilized needles and medical instruments, the reuse of disposable needles and syringes, and contamination of multiple dose medication vials are still responsible for transmission which has made it a significant problem.[40][41][42][43]

There are also some cases of occult HBV infections which are described as the existence of HBV DNA without detectable HBsAg. Antibodies against hepatitis B core antigen (anti-HBc) are often the only serum markers in mono-infected patients with occult HBV. So in cases with high risk of hepatitis B where HBsAg is negative, it is needed to check anti-HBc level. The presence of anti-HBc is rarely coupled with occult infection in HIV-uninfected patients, though. Thus, HBV DNA should be measured.[44]

### Co-Infection with HIV

HBV and HIV have similar characteristics such as transmission modes; using a reverse transcriptase enzyme in replication; the tendency to develop chronic infections, which are often difficult to treat; and finally, an immense capacity of mutation in their genome, causing rapid emergence of mutant strains, some of which are resistant to widely used anti-viral agents. In addition, both viral genomes can integrate within the host genome, a process which is obligatory for the life cycle of HIV, but not for HBV.[45]

Among the estimated 40 million persons infected with HIV worldwide, an estimated 2–4 million are chronically infected with HBV. [31]In general, HBV tends to be more aggressive in HIV-positive individuals than in mono-infected individuals, with higher HBV carrier rates, higher levels of HBV viremia, more frequent episodes of activation, and faster progression to cirrhosis.[46][47] Several factors are effective in estimating these co-infection rates, such as geographic differences in the incidence of chronic infection by age, the efficiency of exposures which is directly responsible for most transmissions, and the prevalence of persons at high risk for infection.[36] Coinfection with HBV and HIV follows a different course everywhere in the world. For example, neonatal or childhood infection, with either vertical or horizontal transmission is the most common route of transmission in Africa and Asia after birth. In parts of Africa, ritual scarification seems to have a major role in the adolescent transmission of HBV.[48][49] (ritual scarification is known as the practice of making small incisions in the skin of adolescents and rubbing black ash in the wounds to form scars; the instruments being used for cutting are not sterilized between rituals).

Co-infection with HIV alters the natural history of chronic hepatitis B. Increasing serum HBV DNA concentrations, decline in liver enzyme levels and developing faster liver cirrhosis, particularly in those with low CD4 counts, are some of the symptoms of this phenomenon (Table 2). The most prominent issue among all these negative consequences is the faster progression of HBV-related liver disease. Cirrhosis has been more commonly reported in HIV-HBV coinfection individuals despite lower alanine aminotransferase (ALT) levels than in HBV mono-infection, and it may be related to lower CD4+ T cell counts.[50][51] Co-infection of HBV with HIV changes the natural history of HBV infection. HIV-HBV coinfected patients seroconvert from HBV e (precore) antigen (HBeAg) to HBV e antibody less frequently and have higher HBV DNA levels but lower levels of alanine aminotransferase (ALT). HBeAg is an accessory protein of HBV and is not necessary for viral replication or infection. Most studies on natural history of HIV- HBV co-infection until now have principally focused on non-Asian patients with HBeAgpositive.[50][52]

**Table 2:** Effects of HIV on the Natural History of Adult-Acquired HBV Infection.[50]

**Effects**	**Comment**
Increase risk for developing chronic HBV infection	Studies in men who have sex with men. Lower CD4+
Decreased rate of HBeAg clearance	T-cell count with higher risk of chronicity
Increased HBV replication	Demonstrated with higher HBV DNA levels
Decreased inflammatory response to chronic hepatitis B	Lower ALT levels
Increased liver disease progression	More cirrhosis and higher liver-related mortality

### Diagnosis

Hepatitis B virus is a dynamic virus and its coinfection with HIV makes the diagnosis process significantly difficult. However, proper diagnosis and monitoring of co-infection of hepatitis B and HIV, along with complete understanding of the mechanisms of drug resistance, will allow researchers and clinicians to manage co-infection cases more efficiently. In general, there is a series of actions for the diagnosis or even prevention of co-infections which are mostly recommended to be accomplished in cases of new HIV infections. First, all individuals diagnosed with HIV recently, should be examined for hepatitis B surface antigen (HBsAg) and anti-HBs. Positive HBsAg expresses HBV co-infection. For HBsAg positive patients, HBeAg, anti-HBe and HBV DNA should be tested, otherwise it is recommended to apply HBV vaccination. In addition to a complete history and physical examinations, newly HIV infected patients’ bloods should be screened for complete blood pictures, clotting profiles including prothrombin time, international normalized ratio (INR), alphafetoprotein (AFP) and tests on liver and renal functions. Performing a baseline hepatic ultrasound is the next step.46 Finally, to screen the condition of the liver, it is highly recommended to assess fibrosis using liver biopsy in individuals suspicious of having HBV/HIV co-infection. However, sampling variations, inter-observers’ variability and the risk of complications have considerably limited the liver biopsy.[53][54]

### Treatment

Significance of HBV/HIV co-infection has led to extensive progress in prescription of HBV in HIV-coinfected individuals. Liver injuries are also more common in these patients for whom treatment options have widely developed.

### Interferon

In HBV monotherapy, suppression of viral replication is the aim of therapy. Interferon in form of standard or pegylated may be a proper treatment for chronic HBV infection in patients who have not yet started highly active antiretroviral therapy (HAART) for their HIV.[59] Interferon is limited to patients who tend to seroconvert; in other words, the female patients with high ALT levels, low HBV DNA levels, and positive HBeAg status in whom liver decompensation has not occurred yet. [55][56]It is also a major concern that interferon therapy is just effective with HBV infection not HIV.[55] It is known that alpha interferon which is available in pegylated form is the most effective therapy for HBsAg positive patients. Levy and Grant have stated that using IFN-α once-weekly for about 16 weeks can be influential in treatment trend. Flulike symptoms, psychiatric effects, bone marrow suppression and thyroid dysfunction are some factors which limit the interferon activity.[57]

In a study performed by Matthews, patients receiving interferon therapy were shown to get significantly more improved with pegylated interferon compared to nucleosides and nucleotide analogues alone. After 48 of therapy in these patients, HBeAg seroconversion, HBV DNA suppression and ALT normalization became better.[58]

## Nucleoside and Nucleotide Analogues

Entecavir, a partial inhibitor of HIV replication along with lamivudine are applied to induce the YMDD mutation in HIV polymerase which is an indication of resistance to therapy. Tenofovir is another option to be chosen for resistance mutations in HIV polymerase.[55] In coinfected patients who develop resistance to lamivudine, the recommended treatment is tenofovir plus entecavir (because there is no cross-reactivity between these two agents), tenofovir plus lamivudine or emtricitabine. Some evidences suggest that entecavir resistance is inclined by lamivudine resistance, but the same studies were performed in patients with very high baseline viral loads; the efficiency of entecavir in patients who had low baseline viral loads is unknown. It is assumed that when entecavir is used along with another potent nucleoside analogue in coinfected patients, the sensitivity of HBV would be more durable than when entecavir is used alone as monotherapy.[59]

## Treating HIV and HBV Infections Concurrently

**Fig. 1:**
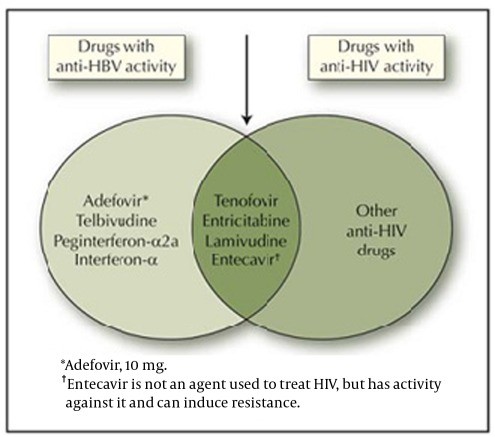
Medications used for treatment of chronic hepatitis B virus (HBV). The arrow refers to medication to use when treatment of both HBV and HIV is considred.[59]

To initiate the treatment in co-infected patients, some factors such as combination antiretroviral therapy for HIV infection, the severity of liver disease, probability of various reactions, and potential adverse events should be taken into account.57 When treatment is necessary for both HBV and HIV infections, HAART is needed for HIV. Using standard therapy for HIV, it is the main treatment strategy in co-infection whereas two effective agents should be selected against HBV infection. Because antiviral resistance is a probability in treatment, choosing active agents seems complicated, that is the choices for treatment of HBV infection are limited by resistance to HIV therapy. The first aim of therapy is to decrease the amount of HBV DNA to an undetectable level; so using less potent agents is not an option. The most potent agent such as tenofovir plus lamivudine or tenofovir plus emtricitabine is recommended as the best choice (Figure 1). [55][56]In a study performed by GERMIVIC Study Group, Piroth et al. reported that lamivudine monotherapy was a very common and a first-line treatment. Other treatment methods included in their study were tenofovir along with lamivudine or emtricitabine, and tenofovir alone.[60] One of the important implications in the treatment of HBV-HIV co-infection was that the dosages of different medications being applied should be adjusted to HBV condition.

## Conclusion

According to the data acquired from different sources and authorities, it is comprehended that HBV/HIV co-infection rate might grow if appropriate controlling mechanisms are not considered. Epidemiological investigations are needed to be performed, so that atypical transmission routes and high risk locations and environment would be identified. Researches in behavior are also necessary in case of further understanding of failures and not effective treatments. Moreover, observation systems seem necessary to monitor infection patterns to target high prevalence regions. To define upper limit of HBV DNA level, clinical studies are needed to be performed for the onset of treatment in both liver disease and co-infection. Finally, recent research showed expanded access to proper treatment methods that can help to reduce the morbidity and mortality rate in HIV-or HBV- infections and also in co-infections, mostly by lowering the viral load among individuals and communities.
